# New trends on childhood nutrition

**DOI:** 10.1186/1824-7288-40-S2-A18

**Published:** 2014-10-09

**Authors:** Luigi Memo, Sonia Viale

**Affiliations:** 1 Paediatric Department, San Martino Hospital, Belluno, Italy; 2 Department of Woman and Child Health, Padua University, Padua, Italy

## Background

An optimal growth is the first objective of feeding during infancy. Recent trials demonstrated that environmental and nutritional influences during critical periods in development, can have permanent effects on an individual's predisposition to diseases in adulthood.

## Materials and methods

This review summarises the studies on the associations between nutrition during pregnancy and infancy with illnesses later in life.

## Results

Maternal nutrition during gestation is important for metabolic programming. Individuals born small for gestational age or prematurely have higher rates of insulin resistance, confirming an association between birth weight and later diabetes, heart disease and obesity [1-3]. High-protein intake during early childhood is associated with obesity, while breastfeeding and timely introduction of complementary foods were shown to protect against obesity in adulthood. Direct benefits of exclusive breastfeeding to the infant's nutrition, gastrointestinal function, host defence and psychological well-being are known in literature. Although evidence is often inconclusive, breastfeeding may be associated with long term benefits such as lower risk of acute illnesses, obesity, cancer, adult coronary heart disease, allergic conditions, type 1 diabetes and inflammatory bowel disease [4].The ESPGHAN recommends complementary foods introduction between 17 and 26 weeks of age [5,6]. Early introduction has been associated with an increased risk of obesity [7,8]; feeding cereals to infants at high risk for type 1 diabetes or celiac disease before 3 months of age may increase the risk of autoimmunity. Later introduction of complementary foods may be associated with adverse effects: decreased growth, iron deficiency, development of atopy and celiac disease or type 1 diabetes. Primary prevention of allergic disease through nutritional interventions has changed [9]. Avoidance diets during pregnancy and lactation are not recommended. Exclusive breast-feeding for at least 4 and up to 6 months is endorsed. Hydrolyzed formula prevents allergic disease and cow's milk allergy in high-risk infants who cannot be exclusively breast-fed. Complementary foods can be introduced between 4 and 6 months of age even for high risk infants. The important role of Vitamin D during pregnancy and infancy is still supported by literature [10,11]. Several recent clinical trials have been conducted to evaluate the effect of supplementation of Docosahexaenoic acid (DHA):it may improve neurodevelopmental outcome in very preterm infants and visual acuity for all infants [12].

## Conclusions

Controlled trials of early nutritional interventions with long-term outcomes are still lacking. Nonetheless, there is ample circumstantial evidence to support clinical efforts to optimize nutrition during gestation, infancy, and early childhood.
